# Game-related injuries in schools: a retrospective nationwide 6-year evaluation and implications for prevention policy

**DOI:** 10.1186/s13584-021-00487-5

**Published:** 2021-08-30

**Authors:** Eli Jaffe, Anna Khalemsky, Michael Khalemsky

**Affiliations:** 1 Magen David Adom, Tel-Aviv, Israel; 2 Hadassah Academic College, Jerusalem, Israel

**Keywords:** School, Injury, Children

## Abstract

**Background:**

Child injury is a global public health problem. Children spend 25–50% of their daytime in school and risks of school accidents are high. The purpose of this study is to perform a comprehensive analysis of game-related injuries.

**Methods:**

A nationwide dataset of 36,002 school injury events that occurred in Israel between 2013 and 2019 and were served by the National EMS, was used. The relations between different variables were demonstrated using multidimensional frequency tables. Z-tests, chi-square tests, ANOVA tests, and J48 classification trees were used to analyze the data.

**Results:**

The prevailing injury cause (36.8%) was “game”, 44.8% of which occur during breaks, and the most frequently injured body regions were head, hand, and leg/foot (47.2%, 26.7%, and 19.7%, respectively). Age was negatively correlated with head injuries and positively correlated with limb injuries. 33% of all injuries occur in the playground and 20.1% occur in the sports field. About 33.3% of game-related injuries in elementary schools occur during the 10:00 a.m. break and an additional 24.7% during the 12:00 p.m. lunch break.

**Conclusion:**

Games are the prevailing cause of school injuries in Israel. Gender and age differences, and seasonal and circadian trends were observed. Understanding the patterns and the trends of school injuries can enable the development of effective prevention policies on the national, municipal, and local levels, focusing the efforts on the key factors affecting injury incidence. Efficient use of resources is necessary, taking into account resource and budget constraints. Efforts can include education of teachers and pupils in relation to school accidents, promoting a safer physical environment, safety education, staff development and family and community involvement, and coordinative training with a focus on proprioception.

**Supplementary Information:**

The online version contains supplementary material available at 10.1186/s13584-021-00487-5.

## Background

The World Health Organization (WHO) considers child injury as a global public health problem. According to an annual WHO report, in 2011 more than 630,000 children were killed by an injury and millions became disabled around the world [1]. In 2018, injuries were among the top causes of death and lifelong disabilities in children aged 5–14 years [2]. The Centers for Disease Control and Prevention (CDC) estimates that between 2001 and 2019 there were about 159 million cases on unintended injuries among children and adolescents (0–19 years old) in the US. In these years, unintentional injuries were the leading cause of death among children and adolescents (1–19 years of age), resulting in 160,823 cases of death. The leading causes of non-fatal injuries were falls (31%), followed by being struck by/against an object (22.2%) and overexertion (9.4%). The leading causes of fatal injuries were traffic accidents (29.5%), followed by drug poisoning (26.2%) and falls (20.3%) [3]. In Israel, the average mortality rate from unintentional injuries in the years 2017–2019, stands at 4.6 per 100,000 children and adolescents up to 17 years old. The leading cause of deaths was car accident (46%), followed by drowning (17%) and suffocation (8%) [4]. In 2015–2017 in Israel, unintentional injures led to a yearly average of 206,000 visits to the emergency room and 26,000 cases of hospitalization (in 2015–2017 the average population of children and adolescents up to 17 years old was 2,397,000) [5].

Children spend 25%-50% of their daytime in school and risks of school-related accidents, are high [6]. Zagel et al. identified school-related injuries as an opportunity for future prevention efforts [7]. Several studies identified sports and physical education accidents as the leading cause of school injuries, followed by school playground activities [6–11]. A systematic review of cohort studies identified male gender, having psychological, behavioral and risk-taking behavior problems, having a large number of siblings, and having a young mother as factors that increase a child's chances of being injured [12]. Several studies reported increased odds of injury for children suffering from attention-deficit hyperactivity disorder (ADHD) [13–15]. Circadian [16] and seasonal [16–18] trends were observed with a higher incidence of injuries in falls, at the beginning of the school year, at the beginning of the week, and one hour before and after lunch.

Upper limbs and in particular hands were identified as the most frequently injured body region, followed by lower limbs and head injuries (6–8,11). Kraus et al. identified the most common types of injuries as contusions (28.5%), distortions (27.5%) and fractures or dislocations (27.4%) [6], Prédine et al.’s results were somewhat different: contusions (50.7%), wounds (18.7%), tendinitis (11.7%), sprains (9.2%), and other (7.3%) [8], and Zagel et al. reported strain/sprain (29.1%), contusion/abrasion (21.7%) and fracture (18.8%) as the three leading types of injuries [7].

The percentage of cases where medical assistance or hospitalization was required varies from study to study, mainly because of different selection criteria used by the authors. Prédine et al. reported that a physician was consulted in 19.5% of the cases and hospitalization was necessary in 2.7% [8]; Chen et al. reported that outpatient medical treatment was required in 23.3% of injuries for female students and 30.1% of injuries for male students while hospitalization was necessary in 3.2% and 4.5% of injuries, respectively [9]; Zagel et al. found that patients were hospitalized in 1.2% of cases [7]; and Kraus et al., who focused on children treated in a hospital, reported a 16% admission rate [6].

The Israeli National Health Insurance Law imposes on the state the responsibility for providing health services to school students [19]. For decades, this service was provided by school nurses, but since 2006 it was gradually privatized. There was a dispute between the Ministries of Finance and Health regarding the privatization, it received much public criticism and was examined by the State Comptroller [20]. Over the years, the service has been provided by a number of contractors and since 2013, the National Emergency Medical Services organization (Magen David Adom or MDA) is responsible for providing primary medical services to all schools and kindergartens (beyond the ambulance services). Schools can request the arrival of medical staff or receive telephone counseling. MDA manages a centralized database of all school injuries and provided anonymized data for this study.

The Israeli Ministry of Education promotes injury prevention policies in schools and kindergarten, including the five-year program “Education to Safety” [21] that promotes partnership with the youth in leading educational and change processes in the community, training and mentoring of staff in the education system, provision of information to the public and more. Circular 134 of the Director General of the Israeli Ministry of Education [22] guides school principals regarding school safety and the prevention of student injuries, including during breaks. For example, teachers on duty are required to prevent dangerous activities and games.

The purpose of the present study is to perform a comprehensive analysis of game-related injuries, in schools in Israel. This type of injuries was chosen because it is a leading cause of injuries in Israeli schools and it is relatively easier to prevent game-related injuries e.g., to forbid some games. The findings of the study could be used to improve safety policy in schools and kindergartens.

## Methods

### Participants

An anonymized dataset of 36,002 injury events was received from MDA. This dataset represents school injury events in kindergartens and schools in Israel assisted by MDA as a primary medical services provider between April 2013 and July 2019. We do not claim that this database covers all school injury events in Israel in the sample period (see details in the Limitations).

### Procedure

For each event the following variables were used:

- Educational stage: kindergarten (3–6 years old), elementary school (6–12 years old), middle school (12–15 years old) and high school (15–18 years old, in some educational programs up to 19 years old). This variable was nominal, and ages provided are for reference only (some overlap is due to differences in the age of the beginning of education).
- Injury cause: game, falling from a height, slipping, violence, animal bite/insect stings, disease. “Game” means that the injury results directly from a game e.g., if a child falls from a height during a game, the injury cause will be “falling from a height”.
- Event location: classroom, hallway, stairway, gym, sports field (usually an open-air basketball/soccer field), playground, outside the school (not on the school territory).
- Event category: before or after school, break (refers both to recess and lunch break), lesson, physical education.
- Injured body region: back, chest, buttocks, foot/leg, groin, hand (includes arm), head, neck, shoulder, stomach.
- Gender: boy, girl.

Due to manual filling of the event reports, a significant amount of data was missing for many variables. In these cases we provided the value “not reported”. The results include all the data, but in some cases in the discussion section we compare the results with and without the missing data.

In order to characterize the structure of the missing data and its effect on the rest of the data and the trends discovered in the study, we performed Little's MCAR test (missing completely at random) using SPSS package [23]. The null hypothesis is that the missing values are distributed completely randomly and not according to any pattern. The alternative hypothesis is respectively that there is a certain pattern in missing values distribution. The null hypothesis was not rejected at a 5% significance level. This finding justifies the following use of missing data in the analysis. The multiple imputations of the missing data were not performed considering a large number of complete observations. The literature supports the decision-making and the researcher's discretion regarding the multiple imputations (or the use of other alternatives for data completion) [24, 25].

**Fig. 1:**
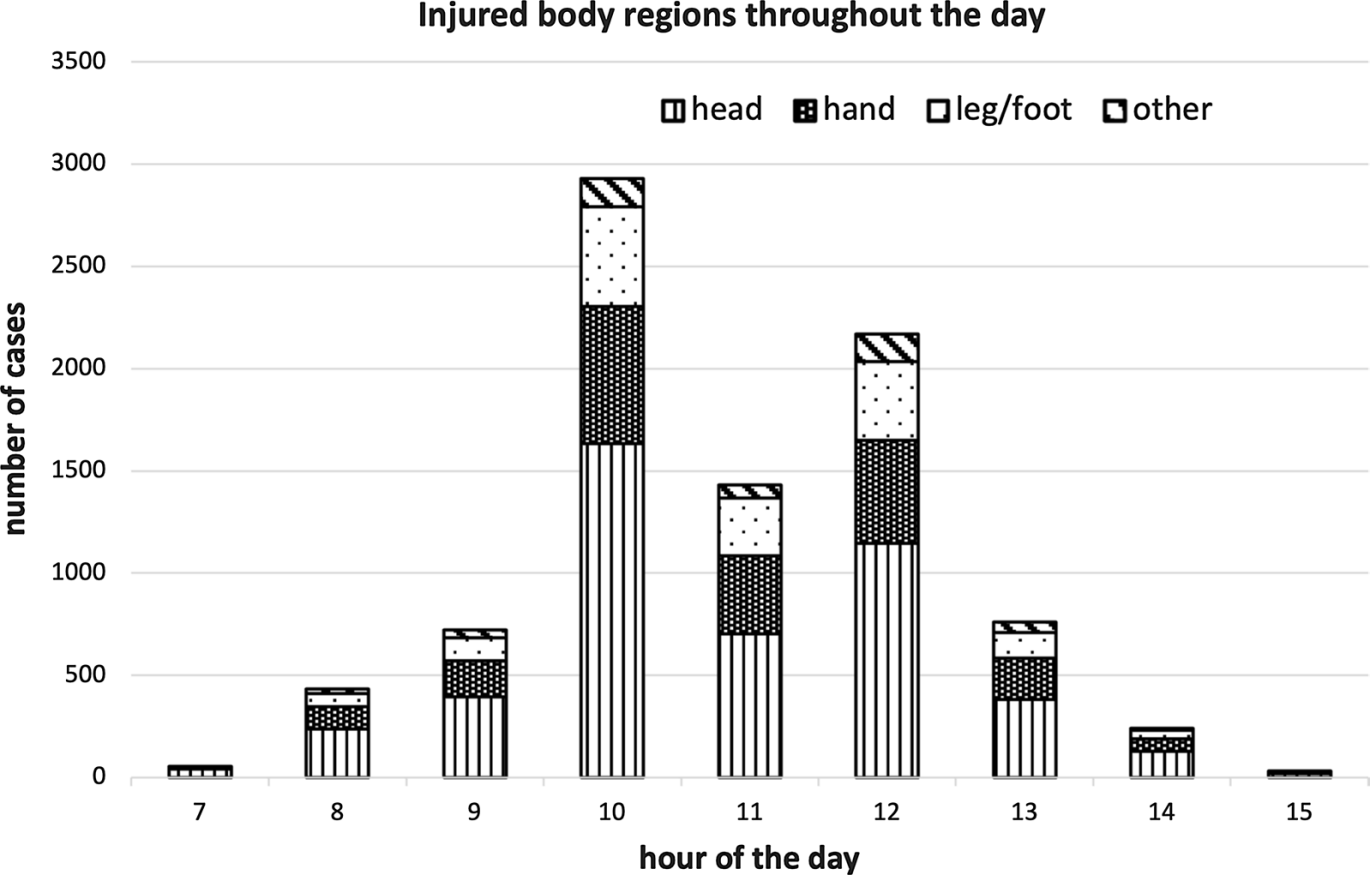
Injured body regions throughout the day

For the analysis of different injury causes we used the full dataset of 36,002 school-injury events (see Table 1). The leading cause of injury was “game” and further analysis focused on these 13,253 events (see Tables 2, 3 and Additional file 1: Table 1). Figure 1 is based on 8852 game-related injury events that occurred in elementary schools.

Given that most variables are categorical, the relations between different variables were demonstrated using multidimensional frequency tables with the number of events in each intersection between three variables and its percentage in each dimension. These tables each provide multiple comparisons to enable pattern and trend detection (see Tables 1 and 2). The only continuous variable in the research is the child's age.

The school year in Israel starts on September 1 and ends in June. However, there are long vacations in September or October (Jewish New Year, Yom Kippur, and Sukkot), in December (Hanukkah), and in March or April (Passover), with the exact date changing every year according to the Jewish lunar calendar. For this reason, a simple analysis of the incidence of injuries for each month would be misleading. We calculated the rate of injuries per school day per month.

### Data analysis

Independent samples two-tailed Z tests were used to investigate the differences between injury cause rates for different educational stages and between body region rates for different educational stages. Rate differences with *p*-values < 0.05 were reported. Chi-square tests of independence at the 5% significance level were used to determine whether there is a significant relationship between an injured body region and an event location, and the results are presented in Additional file 1: Table 1. One-way analysis of variance (ANOVA) tests at the 5% significance level were used to check differences in ages of children between different variable values over the three main categorical variables: event category, body region and injury cause. In addition to the final *p*-value results of the ANOVA tests, we demonstrated some descriptive statistics for each variable value (see Table 3). Each ANOVA analysis produced Scheffe’s post hoc test for multiple comparisons at the 5% significance level.

The J48 classification tree is a predictive analytics tool intended to create a model that classifies given cases to one of the existing categories [26]. In the present research, the target variable for the J48 classification tree was the “event category”. The final result of each experiment includes several quality measures used in different data-mining tasks and for different interpretative purposes. One of the most useful measures is “recall” or the true positive rate. Recall (measured in percent or in a 0–1 range) answers the question of how many relevant items are selected in the model’s final product. The presented recall result is the weighted average of recall values calculated for each variable value. We performed the analysis for each educational stage separately and showed the differences between recall values for all event categories.

## Results

Table 1 presents the distribution of different injury causes for different educational stages.

**Table 1:** Distribution of different injury causes for different educational stages

Injury cause	Category	Kindergarten^a^	Elementary school^a^	Middle + high school^a^	All educational stages^a^
Game	Break	115 (33.4%)	4277 (48.3%)	1546 (38.1%)	5938 (44.8%)
	Lesson	39 (11.3%)	814 (9.2%)	652 (16.1%)	1505 (11.4%)
	Physical education	1 (0.3%)	307 (3.5%)	348 (8.6%)	656 (4.9%)
	Before-after school	2 (0.6%)	134 (1.5%)	39 (1%)	175 (1.3%)
	Not reported	187 (54.4%)	3320 (37.5%)	1472 (36.3%)	4979 (37.6%)
	**Total-game**	**344 (30.5%)**^b^	**8852 (37.8%)**^b^	**4057 (35.4%)**^b^	**13,253 (36.8%)**^b^
Falling from a height	Break	14 (33.3%)	301 (44.1%)	99 (34.3%)	414 (40.9%)
	Lesson	8 (19%)	96 (14.1%)	56 (19.4%)	160 (15.8%)
	Physical education	0 (0%)	12 (1.8%)	12 (4.2%)	24 (2.3%)
	Before-after school	1 (2.4%)	26 (3.8%)	5 (1.7%)	32 (3.2%)
	Not reported	19 (45.2%)	247 (36.2%)	117 (40.5%)	383 (37.8%)
	**Total-falling**	**42 (3.7%)**^b^	**682 (2.9%)**^b^	**289 (2.5%)**^b^	**1013 (2.8%)**^b^
Slipping	Break	74 (32.3%)	1916 (42.3%)	813 (37.3%)	2803 (40.4%)
	Lesson	32 (14%)	510 (11.2%)	300 (13.8%)	842 (12.1%)
	Physical education	2 (0.9%)	98 (2.2%)	114 (5.2%)	214 (3.1%)
	Before-after school	6 (2.6%)	157 (3.5%)	63 (2.9%)	226 (3.3%)
	Not reported	115 (50.2%)	1853 (40.9%)	887 (40.7%)	2855 (41.1%)
	**Total-slipping**	**229 (20.3%)**^b^	**4534 (19.4%)**^b^	**2177 (19.0%)**^b^	**6940 (19.3%)**^b^
Violence	Break	17 (51.5%)	501 (43.2%)	323 (40.6%)	841 (42.3%)
	Lesson	4 (12.1%)	178 (15.3%)	122 (15.3%)	304 (15.3%)
	Physical education	0 (0%)	10 (0.9%)	15 (1.9%)	25 (1.3%)
	Before-after school	0 (0%)	24 (2.1%)	27 (3.4%)	51 (2.6%)
	Not reported	12 (36.4%)	448 (38.6%)	309 (38.8%)	769 (38.5%)
	**Total-violence**	**33 (2.9%)**^b^	**1161 (5.0%)**^b^	**796 (6.9%)**^b^	**1990 (5.5%)**^b^
Animal bite/insects stings and disease	Break	10 (37%)	103 (45.4%)	28 (20%)	141 (35.7%)
	Lesson	2 (7.4%)	24 (10.6%)	29 (20.6%)	55 (13.9%)
	Physical education	0 (0%)	2 (0.9%)	5 (3.5%)	7 (1.8%)
	Before-after school	0 (0%)	5 (2.2%)	5 (3.5%)	10 (2.5%)
	Not reported	15 (55.6%)	93 (41%)	74 (52.5%)	182 (46.1%)
	**Total-animals and diseases**	**27 (2.4%)**^b^	**227 (0.9%)**^b^	**141 (1.2%)**^b^	**395 (1.1%)**^b^
Not reported	Break	131 (28.9%)	2453 (30.9%)	1044 (26.1%)	3628 (29.2%)
	Lesson	34 (13.4%)	998 (12.6%)	663 (16.6%)	1695 (13.7%)
	Physical education	2 (0.4%)	139 (1.7%)	139 (3.5%)	280 (2.3%)
	Before-after school	1 (0.2%)	173 (2.2%)	91 (2.3%)	265 (2.1%)
	Not reported	286 (63%)	4188 (52.7%)	2069 (51.6%)	6543 (52.7%)
	**Total-not reported**	**454 (40.2%)**^b^	**7951 (34.0%)**^b^	**4006 (35.0%)**^b^	**12,411 (34.5%)**^b^
All causes	**All categories**	**1129 (3.1%)**^c^	**23,407 (65.0%)**^c^	**11,466 (31.9%)**^c^	**36,002 (100%)**^c^

^a^For each combination of injury cause and educational stage, the percentage represents the distribution of the event category

^b^The percentage represents the distribution of injury causes within the educational stage (column)

^c^The percentage represents the distribution of events by the educational stage (row)

We investigated the differences in injury cause rates for all educational stages utilizing independent samples two-tailed Z tests for two proportions. Below are the only differences in injury cause rates with *p*-values < 0.05: for “game” injury cause *p*-value (kindergarten, elementary) = 0.006, *p*-value (elementary, middle&high) = 0.008.

After identifying game as the leading cause of injuries, we focused our analysis on game-related injuries only.

Table 2 presents the distribution of body regions injured during games, for different event categories and for different educational stages.

**Table 2:** Game-related injuries-distribution of injured body regions for different event categories over different educational stages

Injured body region	Category	Kindergarten^a^	Elementary school^a^	Middle + high school^a^	All educational stages^a^
Hand	Break	14 (37.8%)	944 (44.5%)	483 (34.9%)	1441 (40.7%)
	Lesson	3 (8.1%)	241 (11.4%)	242 (17.5%)	486 (13.7%)
	Physical education	1 (2.7%)	95 (4.5%)	134 (9.7%)	230 (6.5%)
	Before-after school	1 (2.7%)	34 (1.6%)	17 (1.2%)	52 (1.5%)
	Not reported	18 (48.6%)	806 (38%)	509 (36.8%)	1333 (37.6%)
	**Total-hand**	**37 (10.7%)**^b^	**2120 (23.9%)**^b^	**1385 (34.1%)**^b^	**3542 (26.7%)**^b^
Head	Break	90 (32.5%)	2380 (50.8%)	571 (44.2%)	3041 (48.7%)
	Lesson	33 (11.9%)	381 (8.1%)	188 (14.5%)	602 (9.6%)
	Physical education	0 (0%)	123 (2.6%)	69 (5.3%)	192 (3.1%)
	Before-after school	1 (0.4%)	67 (1.4%)	12 (0.9%)	80 (1.3%)
	Not reported	153 (55.2%)	1730 (37%)	453 (35%)	2336 (37.3%)
	**Total-head**	**277 (80.5%)**^b^	**4681 (52.9%)**^b^	**1293 (31.9%)**^b^	**6251 (47.2%)**^b^
Leg/foot	Break	8 (44.4%)	702 (46.3%)	372 (34.5%)	1082 (41.4%)
	Lesson	2 (11.1%)	134 (8.8%)	178 (16.5%)	314 (12.0%)
	Physical education	0 (0%)	73 (4.8%)	116 (10.8%)	189 (7.3%)
	Before-after school	0 (0%	28 (1.8%)	9 (0.8%)	37 (1.4%)
	Not reported	8 (44.4%)	579 (38.2%)	403 (37.4%)	990 (37.9%)
	**Total-leg/foot**	**18 (5.2%)**^b^	**1516 (17.1%)**^b^	**1078 (26.6%)**^b^	**2612 (19.7%)**^b^
Other body regions	Break	3 (25%)	251 (46.9%)	120 (39.9%)	374 (44.1%)
	Lesson	1 (8.3%)	58 (10.8%)	44 (14.6%)	103 (12.1%)
	Physical education	0 (0%)	16 (3%)	29 (9.6%)	46 (5.4%)
	Before-after school	0 (0%)	5 (0.9%	1 (0.3%)	6 (0.7%)
	Not reported	8 (66.7%)	205 (38.3%)	107 (35.5%)	320 (37.7%)
	**Total-other regions**	**12 (3.5%)**^b^	**535 (6.1%)**^b^	**301 (7.4%)**^b^	**849 (6.4%)**^b^
All body regions	**All categories**	**344 (2.6%)**^c^	**8852 (66.8%)**^c^	**4057 (30.6%)**^c^	**13,253 (100%)**^c^

^a^For each combination of injured body region and educational stage, the percentage represents the distribution of the event category

^b^The percentage represents the distribution of injury causes for the educational stage (column)

^c^The percentage represents the distribution of events by the educational stage (row)

We investigated the differences in body region rates for all educational stages utilizing independent samples two-tailed Z tests for two proportions. Below are the differences in body region rates with *p*-values < 0.05: for “hand” body region: *p*-value (kindergarten, middle&high) = 0.002, *p*-value (elementary, middle&high) < 0.001; for "head" body region all possible differences between educational stages provided *p*-values < 0.001; for "leg/foot" body region: *p*-value (kindergarten, middle&high) = 0.04, *p*-value (elementary, middle&high) < 0.001.

Figure 1 presents the distribution of game-related injury events in elementary schools throughout the day and the distribution of injured body regions. Each column represents the hour of the day and different colors within each column represent injured body regions.

In Israel, the school week starts on Sunday and ends on Thursday in high schools and on Friday in kindergartens, elementary, and middle schools. The highest incidence of game-related injuries is on Wednesdays (20.6%), followed by Thursdays (19.9%), and the lowest incidence is on Fridays (4.9%—in Israel Friday is the last day of the week and children learn only a few hours), followed by Sunday, Tuesday, and Monday (17.7%, 17.9%, and 18.9%, respectively).

We also examined seasonal trends in game-related injuries. The highest incidence of game-related injuries was observed in November (11.88 cases per school day), which is the first full school month after the holidays in September (9.66) and October (9.57). The injury rate drops in December (9.44, Hanukkah), rises in January and February (10.13 and 10.45, respectively), and drops again in March (9.21), April (8.00, Passover), May (8.83), and June (7.74, high school studies end on June 20).

Additional file 1: Table 1 presents the gender distribution of game-related injuries over different injured body regions and different event locations.

Table 3 presents the comparison of age distribution for different values of event category, body region and injury cause. The table includes the following columns: age average for each variable value, CI (95%), standard deviation, median, and interquartile range.

**Table 3:** Age differences within game-related injuries over different injured body regions and different event locations

Variable	Value	Average age	CI (95%)	Standard deviation	Median	IQR
Event category	Lesson	10.96	10.82, 11.11	2.896	11	9–13
	Physical education	11.70	11.51, 11.89	2.487	12	10–13
	Break	9.98	9.92, 10.05	2.557	10	8–12
	Other	10.12	10.05, 10.20	2.752	10	8–12
Body region	Head	9.34	9.27, 9.41	2.726	9	7–11
	Hand	10.98	10.90, 11.06	2.375	11	10–12
	Foot/leg	11.20	11.10, 11.29	2.450	11	9.5–13
	Other	10.76	10.59, 10.93	2.543	11	9–12
Injury cause	Game	10.23	10.19, 10.28	2.708	10	8–12
	Falling from a height	9.84	9.66, 10.03	2.983	10	7–12
	Slipping	10.18	10.11, 10.25	2.854	10	8–12
	Violence	10.66	10.54, 10.78	2.768	11	8.5–13
	Animal bites/insect stings + disease	10.05	9.72, 10.38	3.304	9	7–12
	Other	10.23	10.20, 10.29	2.938	10	8–12

We explored the differences between average ages in different categories for three variables, demonstrated in Table 3 using ANOVA. We found that *p*-values for each test was less than 0.001. In addition, each ANOVA analysis produced Scheffe’s post hoc test for multiple comparisons at the 5% significance level. The following differences are significant:

Event category: lesson vs physical education (*p*-value = 0.000), lesson vs break (*p*-value = 0.000), lesson vs other event category (*p*-value = 0.000), physical education vs break (*p*-value = 0.000), physical education vs other event category (*p*-value = 0.000).

Body region: head vs hand (*p*-value = 0.000), head vs foot/leg (*p*-value = 0.000), head vs other body regions (*p*-value = 0.000), hand vs foot/leg (*p*-value = 0.015), foot/leg vs other body region (*p*-value = 0.000).

Injury cause: game vs falling from a height (*p*-value = 0.004), game vs violence (*p*-value = 0.000), slipping vs falling from a height (*p*-value = 0.035), slipping vs violence (*p*-value = 0.000), falling from a height vs violence (*p*-value = 0.000), falling from a height vs other causes (*p*-value = 0.004), violence vs animal bite/insect sting and disease (*p*-value = 0.010), violence vs other causes (*p*-value = 0.000).

## Discussion

### Injury cause

As Table 1 shows, the prevailing cause of injury was “game” that accounted for 36.8% of events, followed by “slipping” (19.1%). The majority of game-related incidents (44.8% of all incidents with this cause or 71.8% of incidents with this cause in which the event category was reported) occurred during break, followed by game-related incidents that occurred during classes (11.4% and 18.3%, respectively).

In several previous studies, sports and physical education was reported as a separate cause of injury. Summing up all incidents in Table 1 that occurred during physical education resulted in an incidence of 3.35% across all events and 5.9% across events in which the event category was reported. We notice that additional analysis revealed 1306 events that were reported in the event category ‘lesson’ and the event location ‘gym’ or ‘sports field’. While in many cases, sports facilities can be used for non-sports activities e.g., ceremonies, we must assume that some of these events were mistakenly reported in the event category ‘lesson’ instead of ‘physical education’. Even moving all these events to the category ‘sports-lesson’ would result in an incidence of sports-related injuries of 6.9% across all events and 12.26% across events in which the event category was reported. This finding is consistent with that of Josse et al. [18], who reported “playing” and “informal sports” as the leading causes of school injuries, while differing from findings of Prédine et al. [8], Kraus et al. [6], and Zagel et al. [7] who reported sports and physical education as the leading cause of school accidents (52.8% and 39.6% respectively). We conclude that in our sample, ‘game’ was the leading cause of injury. This finding led us to focus this research on game-related injuries.

In our database ‘slipping’ and ‘falling from a height’ are separate causes of injury, while previous studies usually report a single category of ‘falling’; therefore, comparison to previous studies is limited.

Violence accounted in our sample for 5.5% of injuries. This finding is similar to the incidence of fight-related injuries reported by Molcho et al. (4.1%) in a cross country survey [27] and the incidence of intentional injuries reported by Sosnowska et al. (3.1%) in Poland [10], while being much lower than the incidence of intentional injuries reported by Gore et al. (15%) in Canada [11]. Injuries caused by falling from a height, animal bites and insect stings, and disease were rare (3.9% in total).

### Injured body regions in game-related injuries

As Table 2 shows, body regions that were most frequently injured during games were the head, hand, and leg/foot with an incidence of 47.2%, 26.7%, and 19.7%, respectively. Significant differences were observed between educational stages: for kindergarten children head injuries accounted for 80.5% of cases, followed by hand (10.7%) and leg/foot (5.2%), while for elementary school children the distribution is significantly different (52.9%, 23.9%, and 17.1%, respectively) and this downward trend continues in middle and high school children (31.9%, 34.1%, and 26.6%, respectively).

A similar negative correlation between head injury and age was observed in several studies of head trauma epidemiology [28, 29]. These findings are consistent with that of Kraus et. al who reported an incidence of 28.2% (head), 27.6% (hand) and 26.4% (leg/foot) in a breakroom and 31.1%, 39.7% and 21.7% respectively in a school playground [6], and with the findings of Chen et al. who reported 13.8%, 36.0% and 36.8% respectively, in middle and high school children [9].

### Locations of game-related injuries

As Additional file 1: Table 1 shows, more than a half of all injuries occurred either in the school playground (33%) or in the sports field (20.1%). The latter is frequently used in Israel during breaks. Assuming that the distribution of injury locations over events with an unreported injury location is the same as the distribution over events with a reported injury location would raise these numbers to 43.3% and 26.4% respectively. Classrooms accounted for only 10.1% of all cases and 13.3% of cases with reported injury location. These findings are consistent with those of Sosnowska et al. who reported that about half of all injuries occurred in playgrounds (29.7%) and gyms (20.2%) [10] and Gore et al., who reported even higher values for school playgrounds (42%) and gyms (35%) [11].

### Seasonal and circadian trends in game-related injuries

We observed seasonal and circadian trends in game-related injuries.

Figure 1 shows two peak injury times of game-related injuries in elementary schools: 33.3% occur during the 10:00 a.m. break and an additional 24.7% during the 12:00 a.m. lunch break. Some of the events reported at 11:00 a.m. actually occur during the 10:00 a.m. break and are reported with a delay. This finding is consistent with that of Elberl et al. [16], who reported a similar trend in preschool children.

We found a higher incidence of injuries on Wednesdays and Thursdays. Elberl et al. [16] reported a higher incidence of injuries at the beginning of the week, but comparison with this research is limited because Elberl et al. focused on preschool children only. Further research is needed to investigate our findings. One possible explanation is the fact that in many schools, Wednesday is the longest day.

We found an association between daily injury rate and national and religious holidays: months with fewer school days and more holidays were associated with a lower daily injury rate. Further research is necessary to explore this phenomenon. Fatigue and burnout should be investigated as possible causes. Seasonal differences in injuries were reported in several studies: Zemek et al. observed a higher incidence of injuries in Canada in fall and winter resulting from seasonal activities [17], Josse et al. reported a higher incidence of school injuries in fall [18], and Elberl et al. [16] found a higher incidence of injuries at the beginning of the school year.

### Gender differences in game-related injuries

As can be seen in Additional file 1: Table 1, males are involved in game-related injuries more than females (73.1% vs 26.9% respectively). This finding is consistent with many previous studies [6, 9–11, 29] and can usually be explained by behavioural differences between genders [29].

A drill-down analysis in Additional file 1: Table 1 reveals additional trends: the gender differences are extreme for injuries occurring in the sports field (83.9% males vs 16.1% females) and less drastic for injuries occurring inside the school building, e.g., classrooms, hallways, and stairways. Gender differences are higher for head injuries (76.6% vs 23.4%) than for hand and leg/foot injuries (68.4% vs 31.6% and 70.9% and 29.1%, respectively). Previous studies don’t provide a similar analysis for comparison. We believe that the magnitude of the gender differences in injury locations and injured body regions is associated with gender differences in trauma circumstances and mechanisms, but further research is necessary to explore this finding.

### Age differences in game-related injuries

The incidence of game-related injuries presented in Table 1 varies significantly across educational stages: the incidence is lowest in kindergarten (30.5%), highest in elementary school (37.8%), and somewhat lower in middle school and high school (35.4%). These findings are consistent with trends reported by Kraus et al. [6] and Sosnowska et al. [10]. Table 3 provides additional insights: injuries sustained during break were characterized by the lowest average age (9.98 years) while injuries during physical education were characterized by the highest average age (11.7 years). Head injuries were associated with younger ages (average age of 9.34 years) while lower and upper limb injuries were associated with older ages (average age of 11.2 and 10.98 years, respectively). A similar trend was reported by Kraus et al. [6], who also found that head injuries are associated with yard activities and hand injuries are associated with lessons. We found that 50% of game-related injuries occur in students of 8 to 12 years old (IQR = 4 (8–12) with an average age of 10.23 years).

### Classification of event category

J48 classification tree algorithm was applied to classify injury events under the correct event category (lesson, break etc.). The overall model for all game-related events succeeds to correctly classify 93% of all events that occurred during the break (87% in the kindergarten events, 95% in elementary school events, and 91% in middle and high school events). These very high rates show how much the school break events differentiate from other event categories, to the extent that 93% of all break events have definite characteristics that allowed the model to distinguish them from other events. Other event categories, such as lesson and physical education, have less obvious characteristics, which can be partially explained by the semantic confusion between the two categories (see the discussion on “injury cause” above).

### Improving injury prevention policies

The existing policy of teachers on duty that supervise children's activities during breaks is supported by our findings, that suggest that particular attention should be paid to games on the playground. Since games and physical activity are important for children’s physical, social and emotional development [30], our findings should not be interpreted as a call for preventing children from playing, but rather as a call for creating safe environments for playing in schools.

Significant age-related differences in injured body regions suggest that prevention policies should be adapted for different age groups. For example, high percentage of head injuries among young children requires measures such as safe outdoor playing environment [31] e.g., building playground surfaces of shock-absorbing material [32]. Injuries to the extremities during games are more common in older children and can be reduced by coordinative training with a focus on proprioception [33]. Policymakers should consider integration of this type of training into the physical education program.

Seasonal and circadian trends observed in our study require additional prevention efforts during peaks of injuries, such as the assignment of additional teachers on duty and alternative activities for children.

### Limitations

Medical assistance by MDA is called at the discretion of the school secretary or kindergarten teacher. In some cases, they can report the incident to children’s parents who can take their children to their health provider and the event will not be recorded in the MDA database. Some private schools do not use MDA’s services. Additionally, children who are injured on the way to or from school may seek help from their parents rather than from school. Accordingly, we do not claim that the database that we used for this research covers all school injury events in Israel in the sample period.

## Conclusion

Games are the prevailing cause of school injuries in Israel. Most of these incidents occur in school playgrounds and sports fields, during breaks, especially the 10:00 a.m. break and the 12:00 p.m. lunch break. Months with more school days and days at the end of the school week are characterized by a higher incidence of game-related injuries. Males are at higher risk for injury, especially in school playgrounds. Ages 8–12 years account for 50% of game-related injuries.

Prevention efforts should focus on the aforementioned incidents. These efforts can include group intervention programs [34], education of teachers and pupils in relation to school accidents [6], a safe physical environment, safety education, staff development and family and community involvement [35], and coordinative training with a focus on proprioception [33]. Understanding the patterns and the trends of school injuries can enable the development of effective prevention policies on the national, municipal, and local levels. For example, if most injuries occur in the school playgrounds and in the sports fields on Wednesdays and Thursdays, then a teacher should be there to supervise children while playing games.

## Supplementary Information

**Additional file 1: Table 1**: Gender distribution of game-related injuries over different injured body regions and different event locations.

## Data Availability

The data underlying this article were provided by Israeli Magen David Adom by permission. Data will be shared on request to the corresponding author with permission of Israeli Magen David Adom.

## References

[1] WHO | Child injuries [Internet]. WHO. World Health Organization; 2011 [cited 2020 Mar 28]. Available from: https://www.who.int/violence_injury_prevention/child/injury/en/.

[2] World Health Organization. Mortality among children aged 5–14 years [Internet]. 2019 [cited 2021 Jul 31]. Available from: https://www.who.int/news-room/fact-sheets/detail/mortality-among-children-aged-5-14-years.

[3] Centers for Disease Control and Prevention. Leading causes of death visualization tool [Internet]. 2021 [cited 2021 Jul 31]. Available from: https://wisqars-viz.cdc.gov:8006/lcd/home?lcd=eyJjYXVzZXMiOlsiQUxMIl0sInN0YXRlcyI6WyIwMSIsIjAyIiwiMDQiLCIwNSIsIjA2IiwiMDgiLCIwOSIsIjEwIiwiMTEiLCIxMiIsIjEzIiwiMTUiLCIxNiIsIjE3IiwiMTgiLCIxOSIsIjIwIiwiMjEiLCIyMiIsIjIzIiwiMjQiLCIyNSIsIjI2IiwiMjciLCIyOCIsIjI5IiwiMzAiLCIzMSIsIjMyIiwiMzMiLCIzNCIsIjM1IiwiMzYiLCIzNyIsIjM4IiwiMzkiLCI0MCIsIjQxIiwiNDIiLCI0NCIsIjQ1IiwiNDYiLCI0NyIsIjQ4IiwiNDkiLCI1MCIsIjUxIiwiNTMiLCI1NCIsIjU1IiwiNTYiXSwicmFjZSI6WyIxIiwiMiIsIjMiLCI0Il0sImV0aG5pY2l0eSI6WyIxIiwiMiIsIjMiXSwic2V4IjpbIjEiLCIyIl0sImZyb21ZZWFyIjpbIjIwMDEiXSwidG9ZZWFyIjpbIjIwMTkiXSwibnVtYmVyX29mX2NhdXNlcyI6WyIxMCJdLCJhZ2VfZ3JvdXBfZm9ybWF0dGluZyI6WyJjdXN0b20iXSwiY3VzdG9tQWdlc01pbiI6WyIwIl0sImN1c3RvbUFnZXNNYXgiOlsiMTkiXSwieXBsbGFnZXMiOlsiNjUiXX0%3D.

[4] Falk A. Child mortality report from unintentional injuries in 2020 [Internet]. Beterem; 2021 Jan [cited 2021 Jul 26]. Available from: https://www.beterem.org/wp-content/plugins/pdf-poster/pdfjs/web/viewer.php?file=https://www.beterem.org/wp-content/uploads/2020/12/%D7%93%D7%95%D7%97-%D7%AA%D7%9E%D7%95%D7%AA%D7%94-2020-%D7%9E%D7%A2%D7%95%D7%93%D7%9B%D7%9F-0501.pdf&download=true&print=false&openfile=false.

[5] Falk A, Or D, Khalif E. Injuries among children in Israel - “Beterem” report to the Nation [Internet]. Beterem; 2020 Dec [cited 2021 Jul 26]. Available from: https://www.beterem.org/wp-content/plugins/pdf-poster/pdfjs/web/viewer.php?file=https://www.beterem.org/wp-content/uploads/2020/12/%D7%93%D7%95%D7%97-%D7%A1%D7%95%D7%A4%D7%99-%D7%A4%D7%A8%D7%A7%D7%99%D7%9D-%D7%9E%D7%90%D7%95%D7%97%D7%93%D7%99%D7%9D1.pdf&download=true&print=false&openfile=false.

[6] Kraus R, Horas U, Szalay G, Alt V, Kaiser M, Schnettler R (2011). School-related injuries: a retrospective 5-year evaluation. Eur J Trauma Emerg Surg.

[7] Zagel AL, Cutler GJ, Linabery AM, Spaulding AB, Kharbanda AB (2019). Unintentional injuries in primary and secondary schools in the United States, 2001–2013. J Sch Health.

[8] Prédine R, Chau N, Lorentz N, Prédine E, Legras B, Benamghar L (2002). School-related injuries: incidence, causes, and consequences. Rev Epidemiol Sante Publique.

[9] Chen G, Smith GA, Deng S, Hostetler SG, Xiang H (2005). Nonfatal injuries among middle-school and high-school students in Guangxi. China Am J Public Health.

[10] Sosnowska S, Kostka T (2003). Epidemiology of school accidents during a six school-year period in one region in Poland. Eur J Epidemiol.

[11] Gore GC, Magdalinos H, Pless IB (2004). School injuries and preventive policies and programs. Can J Public Health.

[12] Mytton J, Towner E, Brussoni M, Gray S (2009). Unintentional injuries in school-aged children and adolescents: lessons from a systematic review of cohort studies. Inj Prev.

[13] Shem-Tov S, Chodick G, Weitzman D, Koren G (2019). The association between attention-deficit hyperactivity disorder, injuries, and methylphenidate. Glob Pediatr Health..

[14] Man KKC, Chan EW, Coghill D, Douglas I, Ip P, Leung L (2015). Methylphenidate and the risk of trauma. Pediatrics.

[15] Ruiz-Goikoetxea M, Cortese S, Aznarez-Sanado M, Magallón S, Alvarez Zallo N, Luis EO (2018). Risk of unintentional injuries in children and adolescents with ADHD and the impact of ADHD medications: a systematic review and meta-analysis. Neurosci Biobehav Rev.

[16] Eberl R, Schalamon J, Singer G, Ainoedhofer H, Petnehazy T, Hoellwarth ME (2008). Analysis of 347 kindergarten-related injuries. Eur J Pediatr.

[17] Zemek RL, Grool AM, Rodriguez Duque D, DeMatteo C, Rothman L, Benchimol EI (2017). Annual and seasonal trends in ambulatory visits for pediatric concussion in Ontario between 2003 and 2013. J Pediatr.

[18] Josse JM, MacKay M, Osmond MH, MacPherson AK (2009). School injury among Ottawa-area children: a population-based study. J Sch Health.

[19] The National Health Insurance Law [Internet]. [cited 2021 May 17]. Available from: https://www.nevo.co.il/law_html/law01/036_001.htm#med17.

[20] State Comptroller of Israel. Annual report 60א [Internet]. State Comptroller of Israel; 2010 [cited 2021 May 17]. Available from: https://www.mevaker.gov.il/he/Reports/Report_343/6528b095-7b2a-43d2-a85f-8aeee949bfaf/chap-72.pdf?AspxAutoDetectCookieSupport=1.

[21] The Israeli Ministry of Education. Five-year program “Education to Safety” [Internet]. 2018 [cited 2021 May 17]. Available from: https://meyda.education.gov.il/files/bitachon/Homesh.pdf.

[22] The Israeli Ministry of Education. Circular 134 of the Director General [Internet]. 2018 [cited 2021 May 17]. Available from: https://apps.education.gov.il/Mankal/horaa.aspx?siduri=172.

[23] Little RJ (1988). A test of missing completely at random for multivariate data with missing values. J Am Stat Assoc.

[24] Hughes RA, Heron J, Sterne JA, Tilling K (2019). Accounting for missing data in statistical analyses: multiple imputation is not always the answer. Int J Epidemiol.

[25] Madley-Dowd P, Hughes R, Tilling K, Heron J (2019). The proportion of missing data should not be used to guide decisions on multiple imputation. J Clin Epidemiol.

[26] Breiman L, Friedman J, Stone CJ, Olshen RA (1984). Classification and regression trees.

[27] Molcho M, Harel Y, Pickett W, Scheidt PC, Mazur J, Overpeck MD (2006). The epidemiology of non-fatal injuries among 11-, 13- and 15-year old youth in 11 countries: findings from the 1998 WHO-HBSC cross national survey. Int J Inj Contr Saf Promot.

[28] Rivara FP (1984). Childhood injuries, Iii: epidemiology of non-motor vehicle head trauma. Dev Med Child Neurol.

[29] Berney J, Favier J, Froidevaux A-C (1994). Paediatric head trauma: influence of age and sex. Child’s Nerv Syst.

[30] Ramstetter CL, Murray R, Garner AS (2010). The crucial role of recess in schools. J Sch Health.

[31] British Columbia, Community Care Facilities Branch, British Columbia, Office for Injury Prevention, Westcoast Child Care Resource Centre. Preventing injury in child care settings. Victoria, B.C.: Community Care Facilities Branch; 2003.

[32] MedlinePlus. Preventing head injuries in children: MedlinePlus Medical Encyclopedia [Internet]. [cited 2021 May 17]. Available from: https://medlineplus.gov/ency/patientinstructions/000130.htm.

[33] Greier K, Riechelmann H (2012). Ball sports injuries in school sport and means of prevention. Dtsch Z Sportmed.

[34] Bruce B, McGrath P (2005). Group interventions for the prevention of injuries in young children: a systematic review. Inj Prev.

[35] CDC. School health guidelines to prevent unintentional injuries and violence [Internet]. CDC; 2006 [cited 2020 Apr 14]. Available from: https://stacks.cdc.gov/view/cdc/21064/cdc_21064_DS1.pdf.

