# Supplementary material for: Game-related injuries in schools: a retrospective nationwide 6-year evaluation and implications for prevention policy
Source: Isr J Health Policy Res. 2021 Aug 30;10:51. doi: 10.1186/s13584-021-00487-5 (PMC8404308; doi:10.1186/s13584-021-00487-5)
Supplement: Supplementary file 1 — Additional file 1: Table 1: Gender distribution of game-related injuries over different injured body regions and different event locations. [file 13584_2021_487_MOESM1_ESM.docx]

**APPENDIX 1**

**Table 1: Gender distribution of game-related injuries over different injured body regions and different event locations**

| **Injured body region**  **Event location** | **Gender** | **Head**^a^ | **Hand**^a^ | **Leg/foot**^a^ | **Other**^a^ | **Total**^a^ |
| --- | --- | --- | --- | --- | --- | --- |
| **Classroom** | **Male** | 553 (73.5%) | 212 (61.1%) | 79 (53.7%) | 71 (74.7%) | 915 (68.2%) |
|  | **Female** | 199 (26.5%) | 135 (38.9%) | 68 (46.3%) | 24 (25.3%) | 426 (31.8%) |
|  | **All - classroom** | **752 (12%)**^b^ | **347 (9.8%)**^b^ | **147 (5.6%)**^b^ | **95 (11.2%)**^b^ | **1,341 (10.1%)**^b^ |
| **Hallway** | **Male** | 333 (74.5%) | 99 (60%) | 76 (59.8%) | 40 (78.4%) | 548 (69.4%) |
|  | **Female** | 114 (25.5%) | 66 (40%) | 51 (40.2%) | 11 (21.6%) | 242 (30.6%) |
|  | **All - hallway** | **447 (7.2%)**^b^ | **165 (4.7%)**^b^ | **127 (4.9%)**^b^ | **51 (6%)**^b^ | **790 (6%)**^b^ |
| **Outside the school** | **Male** | 38 (82.6%) | 14 (66.7%) | 9 (64.3%) | 4 (66.7%) | 65 (74.7%) |
|  | **Female** | 8 (14.4%) | 7 (33.3%) | 5 (35.7%) | 2 (33.3%) | 22 (25.3%) |
|  | **Total - outside the school** | **46 (0.7%)**^b^ | **21 (0.6%)**^b^ | **14 (0.5%)**^b^ | **6 (0.7%)**^b^ | **87 (0.7%)**^b^ |
| **Sports field** | **Male** | 842 (86.5%) | 703 (81.3%) | 550 (83.3%) | 146 (84.4%) | 2241 (83.9%) |
|  | **Female** | 131 (13.5%) | 162 (18.7%) | 110 (16.7%) | 27 (15.6%) | 430 (16.1%) |
|  | **Total - sports ground** | **973 (15.6%)**^b^ | **865 (24.4%)**^b^ | **660 (25.3%)**^b^ | **173 (20.4%)**^b^ | **2,671 (20.1%)**^b^ |
| **Gym** | **Male** | 175 (71.7%) | 145 (55.6%) | 133 (64.3%) | 38 (69.1%) | 491 (64%) |
|  | **Female** | 69 (28.3%) | 116 (44.4%) | 74 (35.7%) | 17 (30.9%) | 276 (36%) |
|  | **All- gym** | **244 (3.9%)**^b^ | **261 (7.4%)**^b^ | **207 (7.9%)**^b^ | **55 (6.5%)**^b^ | **767 (5.8%)**^b^ |
| **Stairway** | **Male** | 22 (66.7%) | 5 (71.4%) | 16 (55.2%) | 5 (83.3%) | 48 (64%) |
|  | **Female** | 11 (33.3%) | 2 (28.6%) | 13 (44.8%) | 1 (16.7%) | 27 (36%) |
|  | **All - stairway** | **33 (0.5%)** | **7 (0.2%)** | **29 (1.1%)** | **6 (0.7%)** | **75 (0.6%)** |
| **Playground** | **Male** | 1,765 (75.6%) | 662 (66.1%) | 527 (66.1%) | 168 (70.6%) | 3,122 (71.4%) |
|  | **Female** | 570 (24.4%) | 340 (33.9%) | 270 (33.9%) | 70 (29.4%) | 1,250 (28.6%) |
|  | **All - playground** | **2,335 (37.4%)**^b^ | **1,002 (28.3%)**^b^ | **797 (30.5%)**^b^ | **238 (28.1%)**^b^ | **4,372 (33%)**^b^ |
| **Not reported** | **Male** | 1,059 (74.5%) | 583 (66.7%) | 461 (73.1%) | 156 (69.6%) | 2,259 (71.7%) |
|  | **Female** | 362 (25.5%) | 291 (33.3%) | 170 (26.9%) | 68 (30.4%) | 891 (28.3%) |
|  | **All - not reported** | **1,421 (22.7%)**^b^ | **874 (24.7%)**^b^ | **631 (24.2%)**^b^ | **224 (26.4%)**^b^ | **3,150 (23.8%)**^b^ |
| **All locations** | **Male** | 4,787 (76.6%) | 2423 (68.4%) | 1,851 (70.9%) | 628 (74.1%) | 9,689 (73.1%) |
|  | **Female** | 1,464 (23.4%) | 1,119 (31.6%) | 761 (29.1%) | 220 (25.9%) | 3,564 (26.9%) |
|  | **All genders - all locations** | **6,251 (47.2%)**^c^ | **3,542 (26.7%)**^c^ | **2,612 (19.7%)**^c^ | **848 (6.4%)**^c^ | **13,253 (100%)**^c^ |
| **Cramer's V**  **p-value^d^** |  | 0.105  0.000 | 0.171  0.000 | 0.192  0.000 | 0.133  0.035 | 0.130  0.000 |

^a^ - For each combination of injured body region and event location, the percentage represents the gender distribution between females and males

^b^ - The percentage represents the distribution of event location over the injured body region (column).

^c^ - The percentage represents the distribution of events by the injured body region (row)

^d^ - Cramer's V and p-value chi-square tests performed separately for each injured body region
